# Inebriism, a Pathological and Psychological Study

**Published:** 1885-11

**Authors:** 


INEBRIISM, A PATHOLOGICAL AND PSYCHOLOGICAL STUDY. By T. L. Wright, M. D., member of American Association for the Cure of Inebriates. Columbus, Ohio: William G. Hubbard. 1885.

Attention is called in this volume to the modifications imposed by alcohol upon the human constitution in relation to its more prominent powers and tendencies, and the appeal is made to educated men and women, the formulators and leaders of popular sentiment.

It is a fit occasion just now for the people of this country to study this question in all its bearings, and if it is viewed from a professional stand-point, it must appear that all should abstain from the use of intoxicating drinks in health, so that they may receive the full benefits of them as medicines in sickness. This little book of 222 pages is neatly and tastefully gotten up.

